# Earlier diagnosis of small intestine neuroendocrine tumours (SI‐NETs) through transformation of the South Wales NET service

**DOI:** 10.1111/jne.13486

**Published:** 2024-12-26

**Authors:** Harriet L. Gould, Kapish Amin, Thanos Karategos, Sarah Abbas, Susannah Olive, Mathoorika Sivananthan, Ayeesha Rela, Harriet Reed, Catherine Powell, Janu Navaratnam, Rwth Ellis‐Owen, Patrick Fielding, Dipanjali Mondal, Steve Kihara, Gethin Williams, Carys Morgan, Justyna Witczak, Julie Cornish, Sarah Gwynne, James Horwood, Jared Torkington, Rachel Hargest, Adam Christian, Michael Davies, James Ansell, Mohid S. Khan

**Affiliations:** ^1^ Cardiff University Cardiff UK; ^2^ South Wales Neuroendocrine Tumour Service, Department of Gastroenterology Cardiff and Vale University Health Board Cardiff UK; ^3^ Surgical Directorate Cardiff and Vale University Health Board Cardiff UK; ^4^ Department of Radiology Cardiff and Vale University Health Board Cardiff UK; ^5^ South Wales Neuroendocrine Tumour Service Swansea Bay University Health Board Swansea UK; ^6^ Department of Surgery Royal Gwent Hospital Newport UK; ^7^ Velindre Cancer Centre Cardiff UK; ^8^ Department of Endocrinology Cardiff and Vale University Health Board Cardiff UK; ^9^ Department of Cellular Pathology Cardiff and Vale University Health Board Cardiff UK

**Keywords:** carcinoid, clinical presentation, diagnosis, diagnosis times, gastrointestinal symptoms, investigations, neuroendocrine cancer, neuroendocrine neoplasm, neuroendocrine tumour

## Abstract

Small intestine neuroendocrine tumours (SI‐NETs) are often diagnosed late with a UK median of 3 years and high misdiagnosis rates. Previous studies, largely based on patient surveys, offer little data on improving diagnosis. In 2017, the South Wales NET service underwent a nationally commissioned, systematic transformation, aiming to improve diagnosis through the development of a gastroenterology and surgical referral network, and education of these specialities. This study aims to assess the impact of the transformation on SI‐NET diagnosis times and misdiagnosis rates using accurate hospital data, along with the diagnostic routes and investigations used for SI‐NETs. We retrospectively analysed the hospital records of 224 patients diagnosed with SI‐NETs referred to the South Wales NET service (110 pre‐transformation and 114 post‐transformation). Following the service transformation, there was a significant reduction in diagnosis times from a median of 12.5–5.2 months (*p* < .05), at an earlier stage (cases with metastases reduced from 77% to 62%), and reduced misdiagnosis rates from 40% to 25%. Colonoscopy, used to investigate the presenting gastrointestinal symptoms in 42% of patients prior to diagnosis, identified an abnormality in only 28%, compared with 97% with computed tomography (CT) scans. A gastroenterology and surgical referral network across hospitals may improve diagnosis in SI‐NETs, leading to earlier detection and reducing misdiagnosis rates. Further exploration of GP interactions is needed. Caution is needed following negative colonoscopy in patients with persistent lower gastrointestinal symptoms as this could lead to missed SI‐NET diagnosis if further abdominal imaging is not undertaken.

## Graphical abstract

Patients with small intestine neuroendocrine tumours (SI‐NETs) often face considerable delays in diagnosis. The South Wales NET service underwent a nationally commissioned, systematic transformation aimed at improving diagnosis through education and the development of referral networks. This study explores the diagnostic pathway and investigations undertaken for SI‐NETs using hospital data and investigates the potential impact of the transformation on diagnosis times and misdiagnosis rates.

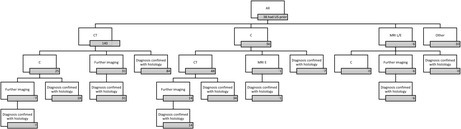

## 1 INTRODUCTION

Neuroendocrine tumours (NETs) are heterogeneous malignancies, arising from neuroendocrine cells distributed throughout various primary anatomical sites. While they are relatively uncommon, with a reported incidence of 8.6 per 100,000 per year in England, their incidence is rising.^1^ Diagnosing these tumours poses a significant challenge, and multiple studies demonstrate considerable delays in diagnosis of up to several years,^2^, ^3^, ^4^, ^5^ during which patients may be transferred between primary and secondary care several times before a diagnosis.^3^

Small intestine NETs (SI‐NETs), which constitute one of the most prevalent subtypes, are particularly difficult to diagnose.^2^ They often present with nonspecific symptoms such as abdominal pain and changes in bowel habit. Furthermore, some remain asymptomatic. A small proportion are functional and can result in carcinoid syndrome. Patients frequently undergo investigations aimed at more common differential diagnoses, such as colorectal cancer and inflammatory bowel disease (IBD). These investigations may include faecal immunohistochemical tests (FITs) and colonoscopy. However, where imaging focuses primarily on the colon, SI‐NETs may go undetected.

Several surveys suggest a large proportion of patients are incorrectly diagnosed before receiving a NET diagnosis, commonly with irritable bowel syndrome (IBS).^2^, ^3^, ^4^ The resulting delays may contribute to poorer patient health‐related quality of life and psychological morbidity.^5^ Therefore, diagnosis times are an important aspect of care to evaluate in NET services. Although several publications have explored clinical presentation, symptoms, and diagnostic delays, these have been based on patient‐reported surveys.^2^, ^3^, ^4^, ^6^ Hospital data in this area are limited, and although delayed diagnosis has been discussed for decades, there is little data on improving times, investigation, and routes of diagnosis.

In 2017, the South Wales NET service, now a European Neuroendocrine Tumour Society (ENETS) Centre of Excellence, responsible for coordinating NET care across South Wales, implemented a new service model as result of national commissioning.^7^ This introduced specialised consultant‐led NET clinics in gastroenterology, oncology and endocrinology; the training of cancer nurse specialists; enhanced NET multidisciplinary team (MDT) meetings and processes and a centralised gastrointestinal‐led model. Importantly, there was greater focus on forming working partnerships with referring specialities through all the region's hospitals (medical, gastroenterology, surgical and specialist nursing) with systematic education on an all‐Wales basis. This has resulted in improvements in patient experience and patient‐reported outcomes.^8^

The primary aim of the study was to evaluate whether diagnosis times and misdiagnosis rates of SI‐NETs improved following the transformation of the South Wales NET service. A secondary aim involved analysing the detailed diagnostic pathway of patients with SI‐NETs in South Wales using hospital records, including the clinical presentation, routes of diagnosis and diagnosing teams. Additionally, it involved exploring the investigations undertaken prior to diagnosis, and their relative sensitivity in diagnosing SI‐NETs (because the presenting symptoms can be indicative of several alternative diagnoses).

## 2 METHODS

Patients identified from the South Wales NET database were included if they had SI‐NETs (jejuno‐ileal NETs or NETs with unknown primary suspected to have an occult small bowel primary). Duodenal and GEP‐NETs from other primary sites, including pancreatic NETs, were excluded. Although evaluating diagnostic improvements similarly in pancreatic NETs could be relevant, they have less severe diagnostic delays than SI‐NETs, with a larger proportion of incidental diagnosis, and follow different diagnostic pathways due to variations in epidemiology, symptom prevalence and hormonal syndromes.^2^, ^9^ Hence, we focused on the homogenous population of SI‐NETs. All patients had a histological diagnosis of NETs, apart from a small cohort where this was not possible or appropriate. Of 224 patients, 110 were diagnosed prior to the service transformation (January 1996 to August 2017); these were selected using a random number generator from patients referred to the NET MDT. A total of 114 patients (diagnosed September 2017 to February 2021) included all patients referred to the service, diagnosed since the transformation. The service evaluation was registered with the Health Board's Service Improvement Committee and approved by the Health Board's Information and Technology department.

The transformation of the service was complex but was planned and commissioned by Welsh Health Specialised Services Committee, clinical experts and patient representatives. In addition to being patient‐centred in its design, it focused on building close working relationships across gastroenterology and surgical specialities from all referring hospitals in South Wales, the main specialities responsible for diagnosing SI‐NETs. Both formal and informal education included teaching at national and regional society meetings, online modules, small group teaching at consultant and trainee level, and individual case interactions.

Data were retrospectively collated from digital hospital records on the Welsh Clinical Portal that holds records on a Wales‐wide basis including primary and secondary care referral letters, discharge letters, outpatient letters, admissions, MDT reports, imaging, histology and endoscopy reports. Symptom onset was estimated using symptom duration stated on outpatient/referral letters. Symptom onset was recorded as the time the first symptom developed, or where there was an obvious change in symptoms in patients with a background of another gastrointestinal condition. Symptoms were considered chronic if they had been present for over 3 months at the time of presentation. Diarrhoea was defined as patient or physician reporting ‘diarrhoea’, faecal urgency, increased stool frequency or change in stool consistency.

Diagnosis date was based on histological diagnosis of a NET on biopsy or resection. Alternatively, if histological diagnosis was not possible, a clinical diagnosis date was based on clinical, imaging, biochemical and nuclear medicine evidence reviewed in the MDT. The speciality team that diagnosed the NET was defined as the referring speciality that the patient was under when the diagnosis was made. The number of specialities a patient saw prior to diagnosis included those seen in outpatient clinic, during emergency admission, or if their case was referred to another non‐NET MDT (each visit was considered an encounter). Chi‐squared test was employed to examine association between categorical variables, with Mann–Whitney test comparing the distribution of continuous variables between independent groups (JASP 0.17, Amsterdam, Netherlands).

An incorrect label of IBS was recorded if this was positively diagnosed and cited in primary or secondary care correspondence or records by a healthcare professional as the reason for symptoms. This included when patients were informed that they had IBS but further investigations undertaken. If another condition such as IBD was thought to explain symptoms, this was also counted as an incorrect label if the NET was found in the same set of investigations and felt to be the more likely cause of symptoms.

## 3 RESULTS

Background characteristics of each group are shown in Table 1.

**TABLE 1:** Background characteristics of pre‐transformation and post‐transformation groups.

	All Patients (*n* = 224)	Pre‐transformation group (*n* = 110)	Post‐transformation group (*n* = 114)	
Gender				
Male	115 (51.3%)	51 (46.3%)	64 (56.1%)	.143
Female	109 (48.7%)	59 (53.7%)	50 (43.9%)	
Age at diagnosis (years)				
Median	64	61	67	<.001**
IQR	56–73	52–69	58–77	
Range	29–90	29–86	36–90	
Grade				
1	137 (61.2%)	72 (65.4%)	65 (57.0%)	.334
2	66 (29.5%)	30 (27.3%)	36 (31.6%)	
3	12 (5.4%)	4 (3.6%)	8 (7.0%)	
Unknown	9 (4.0%)	4	5	
Diagnosis date				
IQR		07/2010–07/2016	06/2018–11/2019	

*Note*: Significance **p < .001.

### 3.1 Diagnosis times

**FIGURE 1:**
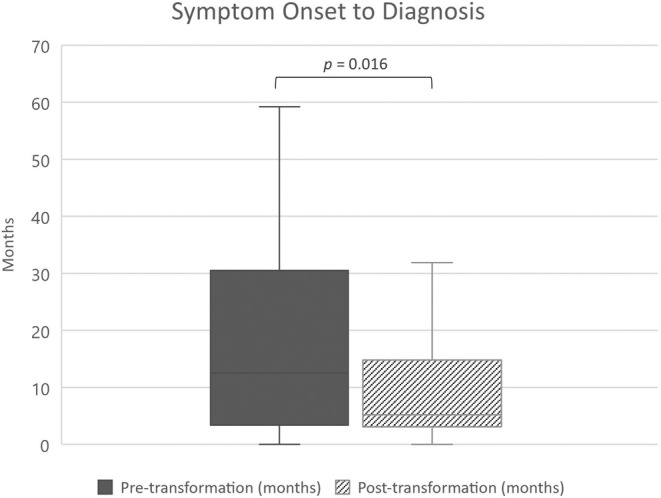
Diagnosis times before and after service transformation for patients with SI‐NETs (*n* = 163). Boxplot demonstrates lower quartile, median, and upper quartile, with range excluding outliers.

For those with symptoms and a clear symptom onset date, there was a reduction in median diagnosis time (from symptom onset, *n* = 163), from 12.5 months (95% CI 8.8–19) pre‐transformation to 5.2 months (95% CI 4.1–6.4) post‐transformation (*p* = .016; Figure 1). Delays in diagnosis over 1 year occurred in 52.6% (40/76) of patients pre‐transformation and 28.7% (25/87) post‐transformation; delays over 3 years occurred in 18.4% (14/76) and 8.0% (7/87) respectively. There was a reduction in the proportion of patients diagnosed with metastases from 77% to 62% (*p* < .05; Table 2). The median time from NET diagnosis to a referral being made to the NET service also reduced, from 31 days (i.q.r: 15–65 days) to 17 days (i.q.r: 6–31 days; *p* < .001).

**TABLE 2:** Comparisons of pathways to diagnosis and proportion of metastatic disease between pre‐transformation and post‐transformation groups prior to correct diagnosis.

	Pre‐transformation group		Post‐transformation group		
	*n*		*n*		
Mislabels (incorrect diagnoses)					
Any mislabel given^a^	85	34 (40%)	114	29 (25%)	.029*
No mislabel given		51 (60%)		85 (75%)	
Discharges from secondary care prior to diagnosis					
None	95	71 (74.7%)	109	88 (80.7%)	.824
Once		20 (21.1%)		18 (16.5%)	
Twice		3 (3.2%)		3 (2.8%)	
Thrice		1 (1.1%)		0 (0%)	
Urgent suspect cancer referral					
Yes	110	7 (6.3%)	114	28 (24.6%)	<.001**
No		103 (93.7%)		86 (75.4%)	
Metastasis at diagnosis					
Yes	100	77 (77%)	101	63 (62.4%)	.024*
No		23 (23%)		38 (37.6%)	

*Note*: Significance *p < .05; **p < .001.

Abbreviations: IBD, inflammatory bowel disease; IBS, irritable bowel syndrome.

^a^ Includes patients given more than one mislabel.

### 3.2 Specialities and routes of diagnosis

The number of patients discharged from a secondary care speciality back to primary care, either with no diagnosis or an incorrect diagnosis (Table 2), at least once, was 25.3% (24/95) pre‐transformation and 19.3% (21/109) post‐transformation. The mean number of specialities that were involved in a patient's care prior to their diagnosis was 1.7 pre‐transformation and 1.5 post‐transformation (range 1–5). Patients had a mean of 3 (range 1–18) encounters with secondary care; this remained unchanged after the transformation.

**FIGURE 2:**
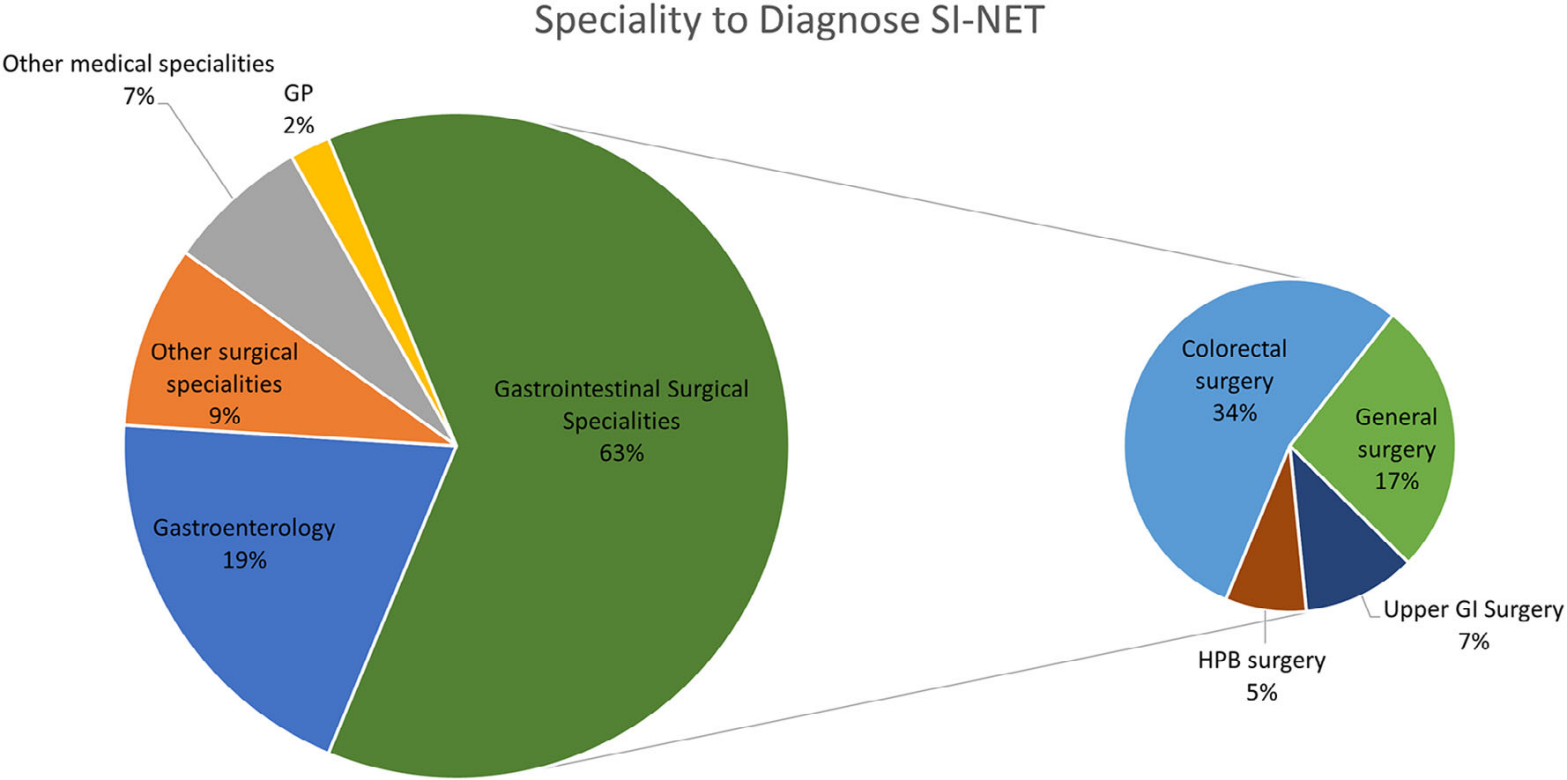
Speciality to diagnose and refer SI‐NET to the South Wales NET service across all years (*n* = 203). Gastrointestinal surgical specialities are divided into surgical subspecialities. SI‐NETs, small intestine neuroendocrine tumours.

The most frequent specialities to diagnose and refer patients to the NET team were gastrointestinal surgical specialities (127/203, 63%), followed by gastroenterology (40/203, 20%; Figure 2). Other surgical specialities included gynaecology, breast, orthopaedic, vascular, urology and ENT. Diagnosis by other medical specialities, including respiratory, haematology, cardiology and endocrinology, was uncommon (7%). In these non‐gastrointestinal specialities, diagnosis was often made as an incidental finding on imaging, or from a metastatic lesion for example, breast biopsy. NETs were identified by GPs who had ordered CT scans prior to referral in 2%.

### 3.3 Incorrect labels

For 199 patients (85 pre‐transformation and 114 post‐transformation), there were sufficient records to confidently determine whether the patient had been given an incorrect label prior to eventual diagnosis. The number of patients given at least one incorrect label reduced from 40% (34/85) to 25% (29/114) after transformation (Table 2; *p =* .029). The most frequent incorrect label was IBS, given to 24.7% (21/85) prior to transformation and 14% (16/114) since. In contrast, a mislabel of IBD was given to 2.4% (2/85) and 3.5% (4/114) respectively. Other incorrect labels included gallstones, diverticular disease, menopause, radiation proctopathy, gastritis, dyspepsia and other cancers.

### 3.4 Symptoms and clinical presentation

**FIGURE 3:**
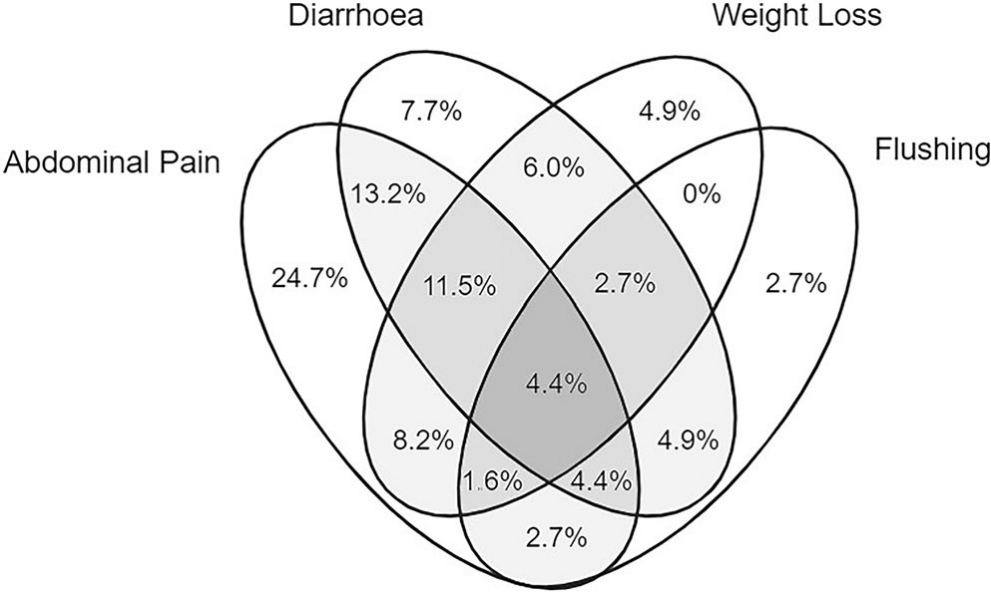
Venn diagram representing all patients with SI‐NETs experiencing any combination of abdominal pain, diarrhoea, weight loss and/or flushing at the time of diagnosis (*n* = 182). Of the total 224 patients, this constituted 81.3% who had one or more of these symptoms. Of the remaining 18.8% (*n* = 42), 13.4% were asymptomatic (*n* = 30), 3.1% had other symptoms only (*n* = 7) and 2.2% were unknown (*n* = 5). Please note that the area is not proportional to the values. SI‐NETs, small intestine neuroendocrine tumours.

Across all years, 194 of the 224 patients (86.6%) had at least one symptom at diagnosis; the most common was abdominal pain (57.6%), followed by diarrhoea (42.9%), weight loss (32.1%), flushing (19.2%), bloating (13.0%) and appetite loss (8.0%). Anaemia was specifically noted in 18 patients (8.0%) as a reason for further investigations. In total, 64 (28.6%) experienced 1 symptom, 52 (23.2%) experienced two symptoms and 78 (34.8%) experienced three or more symptoms. Figure 3 shows the relationship between the most common symptoms.

NET presentation varied. Of 183 patients with complete records, 91 (50%) were diagnosed via secondary care outpatient referral. A further 52 (28.4%) were diagnosed during emergency admissions. Of this latter subgroup, 24 (46%) had a background of chronic symptoms, of which nearly half were already being investigated prior to the emergency admission. Reasons for emergency admission were often due to acute abdominal pain and small bowel obstruction. The proportion of patients diagnosed on emergency admissions remained unchanged after service transformation: 28.7% (25/87) and 28.1% (27/96) respectively. Twenty‐six patients (14.2%) were asymptomatic and diagnosed from incidental findings. A further 14 patients (7.6%) were symptomatic but diagnosed incidentally during investigation for symptoms unrelated to the NET. Scenarios for incidental findings included investigations for staging/surveillance for other cancers (*n* = 10), urinary symptoms (*n* = 7), gynaecological symptoms (*n* = 4), rectal bleeding (*n* = 3), colonic polyps (*n* = 2), chest pain (*n* = 2), trauma (*n* = 2) and bowel cancer screening (*n* = 2). For patients who were concurrently diagnosed with a colorectal cancer (*n* = 5), it was often unclear which cancer was causing symptoms (these were not classed as incidental findings).

### 3.5 Investigations

CT and colonoscopy were the most frequent modalities used to investigate the clinical presentations detailed above prior to diagnosis, often to explore a variety of potential differential diagnoses. The sensitivity of each modality in detecting the SI‐NET and the subsequent action prompted by the findings were analysed as follows.

**FIGURE 4:**
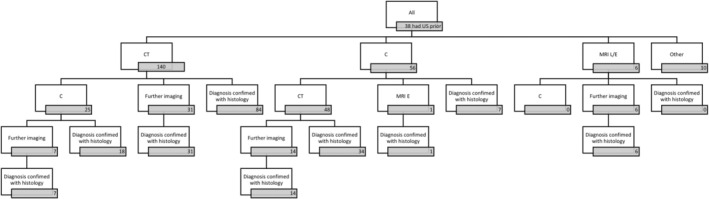
The sequence of investigations undertaken by patients (*n* = 212) in the lead up to diagnosis. The number of patients who underwent an investigation at a given point is stated in the grey box below each label. Further imaging (may be multiple) includes repeat CT, C, MRI E, or MRI L. C, colonoscopy; CT, computerised tomography; MRI E, magnetic resonance enterography; MRI L, magnetic resonance imaging of the liver; US, ultrasound.

Overall, 94.0% of patients (203/216) had an abdominal CT at some point in the lead up to diagnosis. Nearly all (97%) identified an abnormality that prompted further investigation to establish the diagnosis. In comparison, 41.6% (89/214) had a colonoscopy prior to diagnosis that only identified findings raising the suspicion of a NET in 25 patients (28%). Of those with a normal colonoscopy, almost a third (21/64, 32.8%) were discharged following the investigation, leaving the NET undiagnosed. Where full records were available (*n* = 212), the order of all investigations undertaken prior to histological diagnosis is summarised in the flow chart (Figure 4).

As a first‐line investigation, 140 patients (66.0%) had a CT, compared with 56 (26.4%) who had colonoscopy as the first investigation. The radiologist reporting the CT detected an abnormality in 92.9% (130/140) and specifically raised the possibility of a NET in 45.7% (64/140) compared with 20% at colonoscopy (11/56).

As a second‐line investigation following CT, 25/140 (17.9%) patients proceeded to have a colonoscopy. This identified a NET that had not previously been suggested on initial CT in only two cases (8%) (although there were abnormalities on CT). In comparison, 48/56 (85.7%) who had colonoscopy as a first‐line investigation went on to have a CT. The subsequent CT identified an abnormality in 47/48 (97.9%) and specifically suspected a NET in 27/48 (55.1%); only five had been detected at colonoscopy. Other first‐line investigations undertaken included MRI liver, MR enterography, mammography and spinal MRI.

The median time from symptom onset to colonoscopy reduced from 390 to 212 days since the transformation but was not statistically significant (*p* = .384). However, there was a significant reduction in time from symptom onset to abdominal CT scan: 327–103 days (*p* = .004).

## 4 DISCUSSION

There have been several improvements after service transformation, including a significant reduction in diagnosis times by more than half, fewer incorrect initial diagnoses, a greater proportion of patients being diagnosed at an earlier stage and earlier referral to the NET MDT. While international guidelines (ENETS and NANETS) primarily address NET epidemiology, diagnostic tests and management, there is limited information regarding models of care or diagnostic pathways. Our study, from a devolved nation's perspective, provides objective healthcare record data on times and routes of diagnosis in SI‐NETs.

Most diagnoses were made by gastroenterology and gastrointestinal surgery specialities (83%), with primary care contributing only 2%. The transformation in South Wales established networks between these specialities and the central NET service, leading to 1–2 secondary care specialities involved in a patient's diagnosis, over an average of three encounters. This is considerably lower than the 12 encounters reported in patient surveys, where recall bias may lead to overestimation.^6^ Enhanced education and closer working relationships between referring specialities may have improved awareness of SI‐NET presentation, leading to more specialists seeking earlier advice and support, aligning with the British Society of Gastroenterology's strategy.^10^ This is supported by our findings that CT scans were performed earlier after transformation, and there was a reduction in misdiagnosis.

Early diagnosis increases the likelihood of detecting NETs before metastasis occurs. Previous studies have shown that earlier stage at diagnosis is associated with improved prognosis.^1^, ^11^ For example, a large epidemiological study demonstrated 5‐year survival rates of 82% for localised well‐differentiated NETs compared with 35% for those with distant spread.^11^ Our study suggests that the transformation of the NET service resulted in earlier diagnosis from symptom onset, potentially leading to improved prognosis. Additionally, our previous research indicated that this transformation led to improved patient‐reported outcomes and patient experience.^8^ We acknowledge the high rate of metastatic disease at diagnosis in our study (compared with others 30–58%), possibly due to including metastatic disease from unknown primaries.^1^, ^12^ Earlier diagnosis in our study cannot be solely attributed to increased symptoms from more advanced or metastatic disease, as the proportion of patients with metastases decreased after transformation.

Symptom data comparison to previous studies is challenging, but our study suggests a slightly higher proportion of symptomatic patients at 87% compared with 79%–83% in earlier patient surveys.^2^, ^6^ These surveys reported a wide range of duration of symptoms prior to diagnosis, with delays over 3–5 years in a significant proportion of cases (29–34%).^2^, ^3^, ^6^ This difference may be due to our data being less affected by recall bias and the specificity of our healthcare system. We reported a lower diagnosis rate through emergency admission compared with literature (28% vs. 37%).^2^ Although these did not improve through transformation, half had already been investigated as an outpatient. Most patients in our study had multiple symptoms, in contrast to a patient‐based survey where 70% reported only one symptom.^2^ This finding can be attributed to the accuracy of hospital records, which are less affected by recall bias. Our study reveals similar symptoms of abdominal pain, diarrhoea, bloating and flushing as previous studies, but we report anaemia and weight loss as additional features, potentially useful for future guidelines and research. When only one symptom was described, it was commonly abdominal pain, suggesting a need to be aware of NETs when developing local pathways for investigating lower gastrointestinal symptoms.

Misdiagnoses given to patients with NETs, such as IBS, dyspepsia, gallstones, menopause, constipation and IBD, as reported in previous studies, were also common in our study; other labels given included diverticulosis and other cancers.^2^ Our transformation significantly reduced the misdiagnosis percentage from 40% to 25%, compared with the UK figure of 53% reported in the SCAN patient survey.^13^ This improvement is attributed to our network approach, forming relationships with referring hospitals, and staff education, which has been effective in other diseases.^14^

In terms of diagnosis improvements for symptomatic patients, interventions in common cancers like colorectal cancer, where the FIT aids in risk stratification and earlier diagnosis, show promise.^15^ Recently, Okoli et al. published a scoping review from articles on interventions improving diagnosis in symptomatic individuals with common cancers.^16^ Interestingly, effective methods included MDT collaboration and changes to the care pathway, consistent with our transformation process. Our intervention, through a devolved nation commissioning process including patient representatives, aligns with these findings.

We demonstrate that CT is a more sensitive diagnostic tool than colonoscopy for SI‐NET detection. In almost all cases, CT detected an abnormality prompting further investigation, leading to diagnosis. In contrast, colonoscopy frequently missed abnormalities later identified on CT, resulting in more frequent misdiagnoses and patient discharges. Our study also found colonoscopy after CT added little. Nevertheless, to investigate symptoms associated with SI‐NETs, colonoscopy, which was performed in 42% of cases, may still be essential to exclude other more common pathologies such as colorectal cancer or IBD. Therefore, clinicians should remain cautious of SI‐NETs after a normal colonoscopy. CT colonoscopy (CTC) could be used for suitable patients on a lower gastrointestinal pathway if associated with abdominal pain, weight loss, flushing or anaemia. This has important implications for national guidelines, where earlier use of CT/CTC, when appropriate, may lead to earlier diagnosis.^17^ It is noted that the implementation of a ‘symptomatic FIT’ pathway (for patients with lower gastrointestinal symptoms) in Wales occurred after the majority of the cases were diagnosed in this study. Further studies on FIT in NETs are required. Nuclear medicine studies (including octreotide and Ga‐68 dotatate scans) were not considered as these were primarily undertaken for staging purposes once a diagnosis had already been established.

This is the first study to analyse the diagnostic pathways of SI‐NETs using hospital records rather than patient surveys, eliminating selection and recall bias and providing a more accurate picture of symptoms and diagnosis times. Our study's post‐transformation group's median age is similar to SEER data, and we observed an almost equal distribution of male and female patients, making our findings more representative. One survey notes a lack of diversity in respondents, with 45% having higher education levels^6^; our data are not affected by this bias. Retrospective inspection of records is a limitation, with some missing data, particularly in the pre‐transformation period. However, we used a random selection of historic cases to mitigate this and missing data were similar between groups, except for misdiagnosis analysis. The historic range of diagnosis dates in the pre‐transformation group presents another limitation because advances in technology and knowledge over time could potentially contribute to the observed improvements. However, 75% received their diagnosis within a 6‐year period immediately preceding the transformation in 2017, making this less likely.

Another limitation is the absence of primary care data, which could identify any changes in patient behaviour, such as earlier care‐seeking due to bowel cancer awareness campaigns. We did not have access to primary care data, and some patient surveys report multiple GP visits before NET diagnosis.^2^ The number of ‘fast‐track’ referrals for all cancers has increased in the last two decades, potentially contributing to improved diagnosis times.^18^ The study covered a period of the Covid‐19 pandemic, which caused substantial disruption to NET services, including reports of deferred diagnoses, delayed investigation, postponed surgery and reduction in new diagnoses.^19^, ^20^, ^21^, ^22^ Nevertheless, most patients in the post‐transformation group were diagnosed before the pandemic, and all before the easing of restrictions from the second UK lockdown. Therefore, improvements due to urgent referral after pandemic backlogs are unlikely.

Another limitation is that the post‐transformation group was significantly older at diagnosis, although the reason for this and its impact on our results are unclear. Further studies are needed to explore the effect of age, primary care interactions and the implementation of ‘symptomatic FIT’ pathways on diagnosis times.

In summary, our study is one of the first to describe improvements in diagnosis on a population basis using medical records. It suggests that healthcare transformations in secondary and tertiary care can significantly enhance the diagnostic pathway for diagnosing SI‐NETs. This represents a critical step in improving patient outcomes and provides a model for similar improvements in healthcare systems.

## AUTHOR CONTRIBUTIONS

**Harriet L. Gould:** Conceptualization; formal analysis; investigation; methodology; visualization; writing – original draft; writing – review and editing. **Kapish Amin:** Conceptualization; formal analysis; investigation; methodology. **Thanos Karategos:** Data curation; methodology; writing – review and editing. **Sarah Abbas:** Data curation; methodology; writing – review and editing. **Susannah Olive:** Data curation; methodology; writing – review and editing. **Mathoorika Sivananthan:** Conceptualization; investigation; methodology; writing – review and editing. **Ayeesha Rela:** Conceptualization; investigation; methodology; writing – review and editing. **Harriet Reed:** Conceptualization; investigation; methodology; writing – review and editing. **Catherine Powell:** Data curation; methodology; writing – review and editing. **Janu Navaratnam:** Formal analysis; methodology; writing – original draft. **Rwth Ellis‐Owen:** Data curation; methodology; writing – review and editing. **Patrick Fielding:** Data curation; methodology; writing – review and editing. **Dipanjali Mondal:** Data curation; methodology; writing – review and editing. **Steve Kihara:** Data curation; methodology; writing – review and editing. **Gethin Williams:** Data curation; methodology; writing – review and editing. **Carys Morgan:** Data curation; methodology; writing – review and editing. **Justyna Witczak:** Data curation; methodology; writing – review and editing. **Julie Cornish:** Data curation; methodology; writing – review and editing. **Sarah Gwynne:** Data curation; methodology; writing – review and editing. **James Horwood:** Data curation; methodology; writing – review and editing. **Jared Torkington:** Data curation; methodology; writing – review and editing. **Rachel Hargest:** Data curation; methodology; writing – review and editing. **Adam Christian:** Data curation; methodology; writing – review and editing. **Michael Davies:** Data curation; methodology; writing – review and editing. **James Ansell:** Data curation; methodology; writing – review and editing. **Mohid S. Khan:** Conceptualization; data curation; formal analysis; investigation; methodology; project administration; supervision; visualization; writing – original draft; writing – review and editing.

## FUNDING INFORMATION

This study had no funding.

## CONFLICT OF INTEREST STATEMENT

MSK declares that he received speaker fees and advisory board honoraria from Ipsen, Novartis and BMS. The authors declare no other conflicts of interest.

### PEER REVIEW

The peer review history for this article is available at https://www.webofscience.com/api/gateway/wos/peer‐review/10.1111/jne.13486.

## Data Availability

Due to privacy and ethical concerns, we are unable to share individual‐level data. This study was not preregistered.
